# A study protocol for a randomized controlled trial evaluating vibration therapy as an intervention for postural training and fall prevention after distal radius fracture in elderly patients

**DOI:** 10.1186/s13063-019-4013-0

**Published:** 2020-01-16

**Authors:** Ronald Man Yeung WONG, Wing-Tung HO, Ning TANG, Chi Yin TSO, Wai Kit Raymond Ng, Simon Kwoon-Ho CHOW, Wing-Hoi CHEUNG

**Affiliations:** 1 Department of Orthopaedics and Traumatology, Prince of Wales Hospital, The Chinese University of Hong Kong, Sha Tin, Hong Kong SAR, China; 2 Department of Orthopaedics and Traumatology, Prince of Wales Hospital, Hospital Authority, Sha Tin, Hong Kong, China

**Keywords:** Distal radius fracture, Vibration, Fall prevention, Postural stability, Randomized controlled trial

## Abstract

**Background:**

Fractures of the distal radius are one of the most common osteoporotic fractures in elderly men and women. These fractures are a particular health concern amongst the elderly, who are at risk of fragility fractures, and are associated with long-term functional impairment, pain and a variety of complications. This is a sentinel event, as these fractures are associated with a two to four times increased risk of subsequent hip fractures in elderly patients. This is an important concept, as it is well established that these patients have an increased risk of falling. Fall prevention is therefore crucial to decrease further morbidity and mortality. The purpose of this study is to investigate the effect of low-magnitude high-frequency vibration (LMHFV) on postural stability and prevention of falls in elderly patients post distal radius fracture.

**Methods:**

This is a prospective single-blinded randomized controlled trial. Two hundred patients will be recruited consecutively with consent, and randomized to either LMHFV (*n* = 100) or a control group (*n* = 100). The primary outcome is postural stability measured by the static and dynamic ability of patients to maintain centre of balance on the Biodex Balance System SD. Secondary outcomes are the occurrence of fall(s), the health-related quality of life 36-item short form instrument, the Timed Up and Go test for basic mobility skills, compliance and adverse events. Outcome assessments for both groups will be performed at baseline (0 month) and at 6 weeks, 3 months and 6 months time points.

**Discussion:**

Previous studies have stressed the importance of reducing falls after distal radius fracture has occurred in elderly patients, and an effective intervention is crucial. Numerous studies have proven vibration therapy to be effective in improving balancing ability in normal patients; However, no previous study has applied the device for patients with fractures. Our study will attempt to translate LMHFV to patients with fractures to improve postural stability and prevent recurrent falls. Positive results would provide a large impact on the prevention of secondary fractures and save healthcare costs.

**Trial registration:**

ClinicalTrials.gov, NCT03380884. Registered on 21 December 2017.

## Background

Fractures of the distal radius are one of the most common osteoporotic fractures in elderly men and women [1–3] and account for approximately 18% of all fractures in the elderly [4]. Currently, there is approximately a 3:1 female-to-male ratio [2]. These fractures are a particular health concern amongst the elderly, who are at risk of fragility fractures, and they are associated with long-term functional impairment, pain and a variety of complications. Current medical costs for distal radius fractures are estimated to exceed US$535 million each year and projected to rise as the incidence increases [5, 6].

The occurrence of distal radius fractures is well known to be a sentinel event, as these fractures are associated with a two to four times increased risk of subsequent hip fractures in elderly patients [7–9]. This is an important concept, as it is well established that these patients have an increased risk of falling [8]. Fall prevention is therefore crucial to decrease further morbidity and mortality. Studies have shown a significantly increased degree of postural sway in patients with distal radius fractures, in both the anteroposterior and lateral directions, which is strongly characterized in older patients for recurrent falls, and is related to lower limb strength [10]. Another strong association with fragility fractures is sarcopenia, which is loss in muscle mass and strength, leading to postural instability and falls [11]. Recent evidence has shown that the prevalence of sarcopenia reaches up to 95% in male and 64% in female patients after an osteoporotic fracture [12]. The instability of these patients has also been validated with the use of objective measurements from computerized instruments [9].

Despite on-going studies on distal radius fractures, the latest Cochrane systematic review shows a lack of evidence on the effectiveness of current rehabilitation interventions [13]. The American Academy of Orthopaedic Surgeons (AAOS) position statement also recommends patients with fragility fractures to undergo evaluation of osteoporosis and treatment to prevent future fractures [9]. Notably, there are currently no recommendations on the role of balance training or physical conditioning. Consequently, the evaluation and treatment of fall risks have been largely overlooked [14]. Further research that targets rehabilitation and treats postural instability after distal radius fracture to reduce fall rates is therefore warranted.

Low-magnitude high-frequency vibration (LMHFV) is a biophysical intervention that provides non-invasive, systemic mechanical stimulation and was shown to improve muscle strength and balancing abilities in healthy, independent and active elderly women in our previous studies [15, 16]. The device has been proven to act as a form of physical exercise, as muscle activity is induced during vibration [16, 17]. Numerous other studies have reported whole-body vibration to have positive effects on blood circulation in the lower extremities and enhanced muscle performance in the elderly [18, 19]. The use of this device has not yet been established in patients with fractures, but has great potential as a rehabilitation tool. This is the first study to translate LMHFV to distal radius fracture patients, who are high-risk patients prone to falls. This tool would also potentially be more cost-effective.

We hypothesize that LMHFV will improve postural stability and decreases fall rates. As current rehabilitation interventions lack effectiveness, the objective of this study is to investigate the effect of LMHFV on postural stability and recurrent falls in elderly patients post distal radius fracture.

## Methods

### Study design and setting

This study is a randomized, single-blinded controlled clinical trial to evaluate the effect of LMHFV (VH-001 exercise platform; V-health Limited, Hong Kong) on postural stability in elderly patients with distal radius fractures. Patients are recruited from the Prince of Wales Hospital, affiliated with the Chinese University of Hong Kong.

**Fig. 1:**
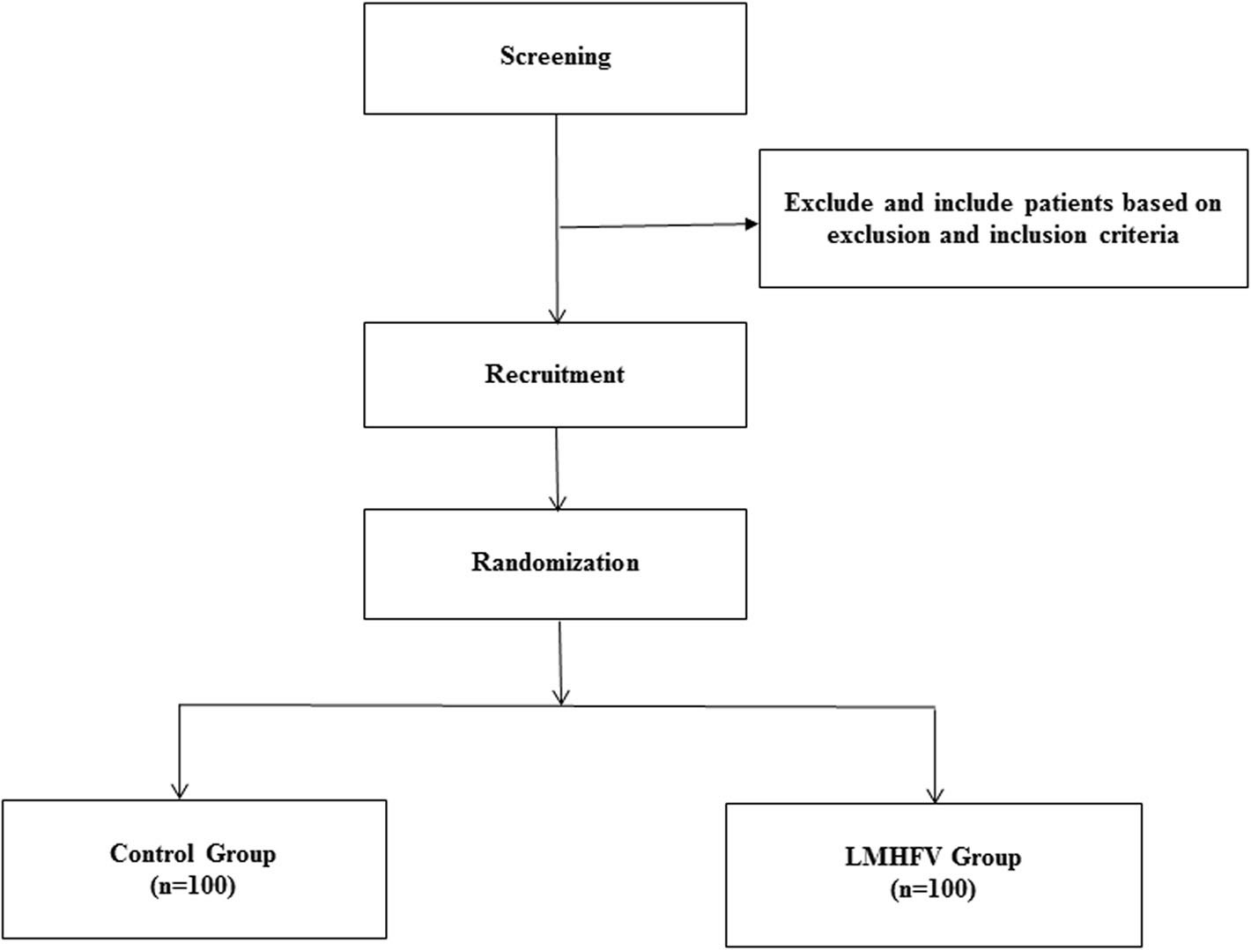
Flowchart of the study design

Figure 1 shows a flowchart of the study design. The Consolidated Standards of Reporting Trials (CONSORT) checklist is provided as Additional file 1; the Standard Protocol Items: Recommendations for Interventional Trials (SPIRIT) checklist is presented in Additional file 2.

### Inclusion criteria

The inclusion criteria are as follows:

1. Aged 60 or above
2. Fracture distal radius after 6 weeks to 3 months
3. Injury was due to unintentional fall.

### Exclusion criteria

The exclusion criteria are as follows:

1. Medical condition causing balance disturbance, e.g. vertigo
2. Participated in supervised regular exercise or physiotherapy for twice a week or more
3. Activities of Daily Living (ADLs) dependent
4. Medications or conditions that affect metabolism of the musculoskeletal system, e.g. hyperthyroidism.

### Sample size

The primary outcome of this study is postural stability. Based on our previous clinical study of LMHFV on balancing ability in normal community elderly [15], we detected a 7.89 mean difference in endpoint excursion (key parameter of balancing ability) between two groups after treatment. A sample size of 85 in each group will have 80% power to detect a significant difference using a two-sided independent *t* test with a 0.05 significance level (PASS 11.0; NCSS Statistical Software, LLC, East Kaysville, UT, USA). Our previous clinical trial also showed a satisfactory compliance rate of LMHFV (averaged 66%) and an approximately 15% dropout rate [15]. Taking account of the dropout, we further increase the sample size to *n* = 100 for each arm (total *n* = 200).

### Recruitment

Eligible patients will be recruited from specialist outpatient clinics or clinical wards consecutively with written consent in the Prince of Wales Hospital, Hong Kong, based on the inclusion and exclusion criteria. Patient demographics on age, gender, education level, ethnicity, occupation, body mass index and smoking and drinking habits will be recorded. Medical history will also be confirmed and recorded from the Clinical Management System (CMS), Hospital Authority, which is the central electronic database for public hospitals in Hong Kong [15]. Before signing the consent form, each patient will be explained the objectives, benefits and risks of the study and their rights and responsibilities, as well as privacy and confidentiality information. An information sheet will be distributed, and all patients are encouraged to ask questions at any time.

### Randomization and blinding

A total of 200 patients will be enrolled. Randomization to either the control or the LMHFV group (*n* = 100 per group) will be performed by envelope drawing of computer-generated random numbers [20] by an independent research staff member. The random number list is kept strictly confidential, and the researchers will not have access to it. The outcome assessor and statistician will be blinded to the group allocation. The central technical staff in our Orthopaedics and Traumatology Department will perform all measurements. The participants will be reminded not to tell the assessor of their allocation. Blinding the patients is not feasible, because the vibration signal from the platform is easily felt, and placebo is rare in vibration clinical trials [15].

### Interventions

Each patient in the LMHFV group will undergo vibration treatment in community centres. We have an established network with LMHFV platforms set up at community centres in several locations in Hong Kong [15, 21]. The patient will stand upright without knee bending on a specially designed vibration platform that provides vertical synchronous vibration at 35 Hz, 0.3 *g* (peak-to-peak magnitude) and a displacement of < 0.1 mm, for 20 min/day at least 3 days/week [18] for 6 months. The research staff will instruct on the safety issues and operative procedures. Each patient in the control group will maintain their habitual life style, and no vibration machine will be used.

### Outcomes and outcome assessments

Outcome assessments for both groups will be performed at baseline (0 month) and at 6 weeks, 3 months and 6 months time points. The primary outcome is postural stability. To assess postural stability, the Biodex Balance System SD (BBS) (Biodex Medical Systems Inc., Shirley, NY, USA) is used to measure the static and dynamic ability of patients to maintain their centre of balance. The score generated by the machine assesses the deviation from centre via an Overall Stability Index (OSI), Anterior/Posterior Stability Index (APSI) and Medial/Lateral Stability Index (MLSI), which have been shown to be reliable tools for objective assessment of postural stability in several studies for elderly patients [16, 22].

Secondary outcomes include the occurrence of falls. To assess the occurrence of falls, patients are required to self-report these events via a fall calendar, which has to be returned at every follow-up visit. Calendar reporting has been well proven to be reliable for fall studies [23, 24]. Other secondary outcomes are quality of life, compliance and adverse events. The health-related quality of life of participants will be assessed with a validated Chinese version of the 36-item Short-Form Health Survey (SF-36). The physical component, mental component and total score will be analysed. All scores range from 0 to 100, with a higher score indicating a better quality of life. In addition, the Timed Up and Go (TUG) test will be used to test basic mobility skills, which are a useful predictor of risk of falls.

Additionally, patients will be phone contacted once every 2 weeks to record any problems in the study.

#### Safety and compliance assessment

A smart card is given to each participant to record and count compliance to the LMHFV device. Any adverse events or problems during the study are recorded by an independent staff member. Any participant may quit the study at any time for any reason; if so, they will be asked whether they wish to be followed up according to the trial schedule.

#### Data collection and management

The reseach assistant will be trained to ensure accuracy of outcome assessments and data collection. The ethics committee will oversee any issues disturbing quality of research, and corresponding measures will be taken if necessary. Patients are free to withdraw from the study at any time without giving any reasons, and their medical care or legal rights will not be affected. The study will comply with the good clinical practice guideline according to the International Council for Harmonisation. Each patient will be assigned an identification code. The patient identification code list and database will be safeguarded.

#### Data analysis plan

Data in this study will be analysed according to the intention-to-treat principle. All results will be expressed as mean ± standard deviation (parametric data). Normality tests will be performed to determine the normal distribution of data. Analysis of variance tests are used to compare means for continuous variables; chi-square tests are used to compare proportions for categorical variables. The statistical analysis will be performed using SPSS 20.0 (IBM, Armonk, NY, USA). The significance level is set at *p* < 0.05 (two-tailed).

## Discussion

Previous studies have stressed the importance of reducing risk of falls after distal radius fractures in elderly patients due to postural instability [25–27]. A prospective, longitudinal cohort study had shown overall functional status and deterioration of mobility for these patients, and future fracture risk increased significantly 1 year after fracture [27]. With the ageing population, prevention of imminent fracture risk, i.e. secondary fractures within 2 years, for patients with distal radius fractures is crucial to decrease mortality and healthcare costs [28].

Numerous studies have proven the effect of vibration therapy in preventing falls and fractures and in improving balancing ability in normal elderly [29, 30]. Our previous randomized controlled trial with 710 healthy, active and independent postmenopausal women over 60 years old had shown LMHFV to have significant improvements in reducing falls, reaction time and movement velocity and in providing maximum excursion of balancing ability assessment as well as quadriceps muscle strength [15]. Our case-control study also showed that at 1 year post intervention of LMHFV, the benefits were retained for the patients [16].

The use of the Biodex Balance System SD (BBS) provides an objective measurement of postural stability and balancing ability for our patients. Several studies have used the device, and it has been proven to be reliable and valid for clinical studies [22, 31–36].

Enrolment in this trial began on November 2018, and completion is expected to take 24 months. The results from this trial would change clinical practice, as currently there are no validated interventions addressing the problem of postural stability for patients with distal radius fractures. We speculate that positive results would allow the incorporation of LMHFV into multidisciplinary rehabilitation programs to improve healthcare for our patients in the future.

### Trial status

At the time of manuscript submission, the trial is still currently recruiting patients.

A table of responses to reviewers’ comments is given in Additional file 3.

## Supplementary information

**Additional file 1.** CONSORT 2010 checklist of information to include when reporting a randomized trial.

**Additional file 2.** SPIRIT 2013 checklist: recommended items to address in a clinical trial protocol and related documents.

**Additional file 3.** Table of responses to reviewers’ comments.

## Data Availability

Not applicable.

## Abbreviations

ADL: Activity of Daily Living
APSI: Anterior/Posterior Stability Index
BBS: Biodex Balance System SD
CMS: Clinical Management System
LMHFV: Low-magnitude high-frequency vibration
LOS: Limits of stability index
MLSI: Medial/Lateral Stability Index
OSI: Overall Stability Index
PWH: Prince of Wales Hospital
TUG: Timed Up and Go (test)
