# Supplementary material for: A study protocol for a randomized controlled trial evaluating vibration therapy as an intervention for postural training and fall prevention after distal radius fracture in elderly patients
Source: Trials. 2020 Jan 16;21:95. doi: 10.1186/s13063-019-4013-0 (PMC6966815; doi:10.1186/s13063-019-4013-0)
Supplement: Supplementary file 3 — Additional file 3. Table of responses to reviewers’ comments. [file 13063_2019_4013_MOESM3_ESM.docx]

**Trials**

**Submission ID: TRLS-D-19-00351**

**Title: Vibration Therapy as an Intervention for Postural Training and Fall Prevention after Distal Radius Fracture in Elderly Patients: A Randomized Controlled Trial**

**TABLE OF RESPONSE** To Reviewers’ Comments

| # | Questions | Response | Changes and Location |
| --- | --- | --- | --- |
| R1.1 | I do not understand why the mechanism of falls, the prevention against falls and the way how to train/impact these mechanisms differ between persons after distal radius fracture and "normal" persons of the same/comparable age, medication and (if applicable) lifestyle. Meta-analytic evidence (doi: doi: 10.1136/bmjopen-2017-018342) on the effects of fall prevention and the effect of vibration exercises on falls is available. Most of the evidence is based on comparisons of vibration therapy against other forms of exercises. Thus, the above mentioned issue is two-fold: Please 1. Provide a rationale for the speciality of and the focus of your study on distal radius fracture patients (why do they differ from a matched "healthy" sample in terms of falls and fall prevention approaches) and 2. Justify why you compare your intervention with a do-nothing control group. As it is known that vibration therapy is helpful, vibration exercises should be compared to falls-preventive exercise without vibration in a superiority trial. Vibration therapy is likely to be more expensive than usual exercises and thus should be more effective when it might be recommended. | We appreciate this important comment.  The prevention against patients with distal radius fracture differs significantly from “healthy” individuals. The reason is due to the concept that these patients have an increased risk of falling and are a high-risk group (1). Studies have shown a significantly increased degree of postural sway in these patients, which is strongly characterized in older subjects for recurrent falls, which is related to the lower limb strength. Another strong association with fragility fractures is sarcopenia, which has loss in muscle mass and strength leading to postural instability and falls (2). Recent evidence has shown that the prevalence of sarcopenia reaches up to 95% in male and 64% in female patients after an osteoporotic fracture (3). Instability of these patients have also been validated with the use of objective measurements including the Dynamic motion analysis (DMA), which is a computerized instrument (4). The fracture cohort showed poorer balance with higher DMA scores at 933 points compared with 790 points for the control cohort (p=0.008). Therefore, the rationale was to treat these high-risk patients, which would potentially be much more cost-effective.  Our intervention was to compare with no vibration therapy. As it is currently not established that vibration therapy is effective in distal radius fracture patients (i.e. the high-risk patients), our initial step was to prove this research question first. Furthermore, despite on-going studies on distal radius fractures, the latest Cochrane systematic review shows a lack of evidence on the effectiveness of current rehabilitation interventions (5). The American Academy of Orthopaedic Surgeons (AAOS) position statement also recommends patients with fragility fractures to undergo evaluation of osteoporosis and treatment to prevent future fractures (4). Notably, there are no recommendations on the role of balance training or physical conditioning. Therefore, in standard clinical practise there is no official treatment for the balance problem in distal radius fracture patients in Hong Kong and many countries. Consequently, the treatment of fall risks in distal radius fracture patients have been largely overlooked (6). Hence, our study design concentrates the use of the current clinical standard as the control for comparison.  We agree that we need to elaborate these details in order to be more clear in our manuscript. We have added the above details and revised our manuscript. | Lines 80-88, 93-94, 103-106, 108-110. |
| R1.2 | In your rationale (abstract and introduction), you write that distal radius fracture often occur in women. You then include both men and women in your study. Please clarify in the rationale if (that) distal radius fractures occur in men, likewise (to what extent). | Thank you for your comment.  Distal radius fracture is one of the most common osteoporotic fractures in middle-aged and older men and women, especially after the age of 50 (7, 8). Overall, there is a 3:1 female to male ratio (8). Both genders are still common to have fracture occurrence due to osteoporosis (4). More importantly, both genders have postural instability after a distal radius fracture. We agree that we need to elaborate these details in order to be more clear in our manuscript. We have added the above details and revised our manuscript. | Lines 40-41, 70-72. |
| R1.3 | Do not begin a sentence with an abbreviation or a number. | We have made sure our manuscript sentences do not begin with an abbreviation or number in the revised version. | Whole manuscript. |
| R1.4 | The term "subject" should not be used when you included participants who subscribed informed consent. Use "participants" or volunteers" or "patients". | We agree with your comment and have changed the term “subject” to “patient”. | Whole manuscript |
| R1.5 | Title: - Unlike in U.S.-American journals, do not use upper case letters, except for the first word - Add "study protocol for a…" | Thank you for your comment. We have revised our title accordingly. | Title |
| R1.6 | Introduction: - lines 73 to 78 are somewhat redundant/superfluous in my opinion. | Thank you for your comment. We have revised the introduction in order to be less redundant. | Lines 74-76. |
| R1.7 | Methods: - In the rationale, an age over 50 is highlighted as a risk factor. Why do you only include patients > 60 years of age? | Thank you for your important comment.  We agree that age over 50 is a risk factor, but our study focuses on treatment on elderly patients. We only include patients 60 years or older based on the general consensus that an elderly patient is defined at this age by the United Nations (UN, 2001). For the World Health Organization (WHO), the commonly used definition of “older person” is associated with the age at which one begins to receive pension benefits (WHO, 2002), which varies in different countries, but most high-income countries use 65 years to define elderly.  In Hong Kong, the official retirement age is 60 (for many years) and recently raises to 65 (<https://www.csb.gov.hk/mobile/english/admin/retirement/183.html>). This helps to justify our recruitment criteria.  The investigators in this study came to a consensus to use 60 years of age as the definition. | N/A |
| R1.8 | - Sample size calculation: 100 - (15 % of 100) = 85, and not 93. Please re-calculate. | Thank you for pointing out our typo mistake. The correct number should have been 85. We have revised our manuscript. | Line 136. |
| R1.9 | Plus: line 154: n = 50? | Thank you for pointing out our typo mistake. The correct number should have been 100. We have revised our manuscript. | Line 154. |
| R1.10 | - Line 217: p <= or < .05? | The p value should have been <0.05. We have revised our manuscript. | Line 216. |

**References**

1.	Kelsey JL, Prill MM, Keegan TH, Tanner HE, Bernstein AL, Quesenberry CP, Jr., et al. Reducing the risk for distal forearm fracture: preserve bone mass, slow down, and don't fall! Osteoporos Int. 2005;16(6):681-90.

2.	Fujita K, Kaburagi H, Nimura A, Miyamoto T, Wakabayashi Y, Seki Y, et al. Lower grip strength and dynamic body balance in women with distal radial fractures. Osteoporos Int. 2019;30(5):949-56.

3.	Wong RMY, Wong H, Zhang N, Chow SKH, Chau WW, Wang J, et al. The relationship between sarcopenia and fragility fracture-a systematic review. Osteoporos Int. 2019;30(3):541-53.

4.	Louer CR, Boone SL, Guthrie AK, Motley JR, Calfee RP, Wall LB. Postural Stability in Older Adults with a Distal Radial Fracture. J Bone Joint Surg Am. 2016;98(14):1176-82.

5.	Handoll HH, Elliott J. Rehabilitation for distal radial fractures in adults. Cochrane Database Syst Rev. 2015(9):CD003324.

6.	Khazzani H, Allali F, Bennani L, Ichchou L, El Mansouri L, Abourazzak FE, et al. The relationship between physical performance measures, bone mineral density, falls, and the risk of peripheral fracture: a cross-sectional analysis. Bmc Public Health. 2009;9.

7.	Øyen J, Diamantopoulos AP, Haugeberg G. Mortality after distal radius fracture in men and women aged 50 years and older in southern Norway. PLoS One. 2014;9(11):e112098.

8.	Wong RMY, Ho WT, Wai LS, Li W, Chau WW, Chow KS, et al. Fragility fractures and imminent fracture risk in Hong Kong: one of the cities with longest life expectancies. Arch Osteoporos. 2019;14(1):104.
